# Effectiveness of Enzyme Dentifrices on Oral Health in Orthodontic Patients: A Randomized Controlled Trial

**DOI:** 10.3390/ijerph16122243

**Published:** 2019-06-25

**Authors:** Hsin-Chung Cheng, Hao-Ting Hu, Ya-Chu Chang

**Affiliations:** 1 School of Dentistry, College of Oral Medicine, Taipei Medical University, Taipei 11031, Taiwan; m204105006@tmu.edu.tw; 2 Orthodontic Division, Department of Dentistry, Taipei Medical University Hospital, Taipei 11031, Taiwan; m8809005@hotmail.com

**Keywords:** enzyme, fluoride, dentifrice, orthodontic patient, dental care, oral health literacy

## Abstract

Plaque accumulation and white spot lesions are common adverse effects of fixed orthodontic appliance use. This study compared the effects between enzyme-containing and conventional dentifrices on orthodontic patients. This double-blind randomized controlled trial included 42 orthodontic patients (25 women and 17 men: 22.7 ± 4.2 years) from Taipei Medical University Hospital between 2017 and 2018. The patients were randomly divided into three groups and assigned to dentifrice use during the first 3 months of the orthodontic treatment: group 1 used dentifrices containing enzymes including amyloglucosidase and glucose oxidase, group 2 used dentifrices containing 1450 ppm fluoride, and group 3 used natural dentifrices containing no chemical agent. White spot lesion index (WSL), gingival bleeding index (GBI), and visible plaque index (VPI) were recorded and analyzed. WSL, GBI, and VPI values exhibited no significant difference among the three groups. WSL increased significantly in group 3, GBI decreased significantly in all groups, and VPI decreased significantly in groups 1 and 2. No significant difference was observed between the use of enzyme-containing and conventional dentifrices after fixed orthodontic appliance placement.

## 1. Introduction

Despite advances in orthodontic treatment, some adverse effects are generally observed, including decalcification of the enamel, also known as white spots lesion, and gingival problems [1]. Moreover, orthodontic treatment with fixed appliances can induce an increase in dental plaque volume.

Various conventional strategies have been developed for preventing plaque-induced diseases, including mechanical elimination of plaque, use of fluoride-containing oral care products, and use of biocides with chlorhexidine [2]. However, the concentrations and frequency of use of fluoride and antiseptics should be limited to prevent side effects, such as acute and chronic toxic effects to the skeletal, gastrointestinal, renal, and central nervous systems that are potentially associated with exposure to various levels of fluoride [3], and causing discoloration of teeth and tongue, taste alterations, and gene mutations during chlorhexidine use [4,5].

With the increasing attention to the innate host defense system, more minimally invasive and human-friendly therapies have been considered, such as the use of enzyme-containing formulas [2,6,7], probiotic consumptions [8], and plant extracts [9,10]. The innate defense factors in human saliva are peroxidase enzymes, lysozyme, and lactoferrin. These proteins can limit bacterial or fungal growth, interfere with bacterial glucose uptake or glucose metabolism, and promote bacterial aggregation and elimination [11]. Since 1973, numerous clinical and laboratory trials have explored the effectiveness of the natural lactoperoxidase system by adding several enzymes at various combinations to dentifrices and mouth rinses [7].

Whether enzyme-containing dentifrices provide more beneficial effects than conventional dentifrices remains inconclusive. Numerous studies have evaluated the effects of dentifrices on white spot lesions and plaque accumulation. Some studies have indicated additional benefits of enzyme-containing dentifrices [6,7,12,13], whereas some have demonstrated no differences in benefits between enzyme-containing and conventional dentifrices [14,15].

Among patients with fixed orthodontic appliances, fluoride products produce a higher degree of protective effects on white spot lesions, plaque accumulation, and gingival health [16,17,18]. However, no study has compared the effects between enzyme-containing dentifrices and conventional dentifrices on orthodontic patients.

Therefore, this study (1) compared the effects of enzyme-containing dentifrices and conventional dentifrices on orthodontic patients, and (2) evaluated user satisfaction regarding enzyme-containing dentifrices and conventional dentifrices.

## 2. Materials and Methods

### 2.1. Ethical Procedures and Informed Consent

This study was conducted in accordance with the Declaration of Helsinki, and the protocol was approved by the Research Ethics Committee of the Taipei Medical University Joint Institutional Review Board (date of approval: April 20, 2016; approval no.: TMU-JIRB No. N201601017). All participants signed the informed consent form for inclusion before enrollment in the study.

### 2.2. Study Design and Participants

This was a 3-month-long, double-blind, randomized controlled trial. The participants were patients undergoing full-mouth orthodontic treatment in the orthodontic department of Taipei Medical University Hospital from 2017 to 2018. Patients who fulfilled the following criteria were included in the study: (1) having permanent dentition, including severe crowding or spacing, (2) undergoing full-mouth complete orthodontic treatment with fixed appliances, and (3) aged > 18 years. Patients with severe periodontitis or caries and aged <18 years were excluded. After screening, block randomization was used, and the patients were randomly allocated to three groups through selection using sequentially numbered (1, 2, and 3), opaque sealed envelopes at a ratio of 1:1:1.

### 2.3. Dentifrices

The dentifrices assessed are listed as follows: group 1’s dentifrices contained enzymes including amyloglucosidase, glucose oxidase, lactoperoxidase, lysozyme, and lactoferrin (Intelligent^®^, Free Bio-Technology Corp., Taipei, Taiwan); group 2’s dentifrices contained 0.315% *w*/*w* sodium fluoride (i.e., 1450 ppm fluoride) (Sensodyne^®^ ProNamel^®^, GlaxoSmithKline Consumer Healthcare, Brentford, U.K.); and group 3’s dentifrices contained naturally derived ingredients without fluoride (no added chemical agents) (Jack N’ Jill LLC, Carson, CA, USA). Group 3 was the control group. All dentifrice tubes were wrapped with white tape, such that they appeared similar from the outside and were coded with the numbers 1, 2, and 3. The code for each dentifrice was placed in a sealed opaque envelope and kept in a locker of the orthodontic department office.

### 2.4. Assessments

The following clinical data were recorded: white spot lesion index (WSL) (Gorelick et al., 1982) [19], gingival bleeding index (GBI), and visible plaque index (VPI) (Ainamo and Bay, 1975) [20].

For WSL, the labial and buccal surfaces of all bonded teeth were visually examined and registered as follows:

1. No white spot formation
2. Slight white spot formation (thin rim)
3. Excessive white spot formation (thicker bands)
4. White spot formation with cavitation

For GBI, a 0.5-mm-diameter periodontal probe was used for gentle probing of the orifice of gingival sulcus, and only the gingival margin at the mesiolabial surfaces was evaluated. Bleeding was scored as 1, and no bleeding within 10 s was score as 0.

For VPI, visible plaque was scored as 1, and nonvisible plaque on the mesio-buccal surface of every bonded tooth after rinsing and drying of the tooth surface was scored as 0.

The five timepoints for scoring were baseline (T0, before orthodontic appliance placement), 3 weeks of treatment (T1), 6 weeks of treatment (T2), 9 weeks of treatment (T3), and 12 weeks of treatment (T4).

### 2.5. Questionnaire Design

The first questionnaire was administered to every patient at baseline. The questionnaire evaluated tooth brushing habits and oral conditions of the patients. It comprised questions on brushing frequency and brushing time under the brushing habits category. Symptoms including oral ulceration, gum bleeding, tooth sensitivity, dry mouth, and bad breath were to be self-assessed under the oral condition category. After the experiment, the second questionnaire was administered, which also evaluated tooth brushing habits, oral condition, user satisfaction, and improvement after using the dentifrices during 12 weeks of treatment.

### 2.6. Trial Flow

After baseline data collection, all teeth were cleaned using a low-speed nylon brush. The self-ligating brackets (Damon 3MX, Ormco, Glendora, CA, USA) were directly bonded from incisors to premolars, and no other orthodontic attachments, intraoral elastics, or TADs (temporary anchorage devices) were placed during the trial. The bands were fitted and cemented in both first and second molars. Thereafter, the patients were randomly allocated into three groups. The corresponding dentifrices and oral hygiene instructions were given to every patient. All patients were instructed to brush their teeth for 3 min with the dentifrices four times a day (after three meals and before sleep). They were instructed to use 1 cm of the dentifrice each time and brush without diluting the dentifrice in water. The patients were called for follow-up every 3 weeks in the first 3 months of the orthodontic treatment, and the measurements were recorded before the orthodontic adjustment during the follow-up visits.

### 2.7. Statistical Analyses

Statistical analyses were performed using SigmaPlot (version 14.0; Systat Software, San Jose, CA, USA). The data were tested for normality (Shapiro–Wilk test) and homogeneity of variance (Brown–Forsythe test). The Kruskal–Wallis test was used to compare the nonparametric variables among the three groups at baseline. The Friedman test was used to compare the nonparametric variables, one-way repeated measures analysis of variance (ANOVA) was used to compare the parametric variables within each group during the experiment, and Tukey’s post hoc test (*p* < 0.05) was used in all pairwise multiple comparisons. The McNemar’s test, chi-square test, and Yates continuity correction were applied for comparisons among the questionnaires. The significance level was set as *p* < 0.05 for all statistical tests. The confidence interval was 95%.

## 3. Results

### 3.1. General Description

**Figure 1:**
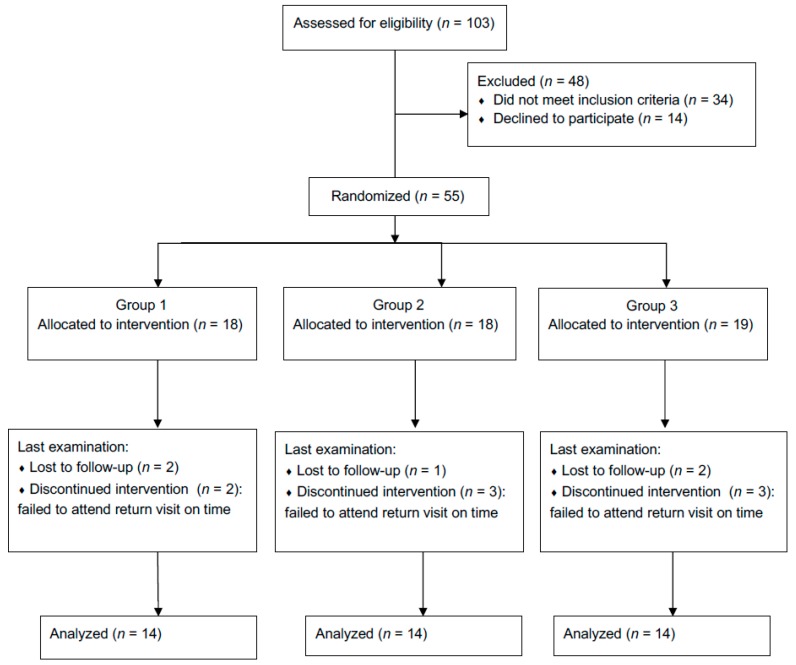
Flow of study phases.

For patient recruitment, 103 patients were assessed for eligibility. In total, 69 patients fulfilled the inclusion criteria, of which 55 patients volunteered to participate in the trial. Among the volunteers, 42 completed the 3-month experiment and 13 were lost to follow-up (Figure 1). The dropout rate for the subject numbers was similar among the three groups and ranged from 22% to 26%. Finally, 42 patients were analyzed, including 25 women and 17 men, with a mean age of 22.7 ± 4.2 years. The distribution of age and sex was statistically similar among the three groups. Revelation of the assigned codes in March 2019 showed the following: group 1 contained 14 patients who used enzyme-containing dentifrices, group 2 contained 14 patients who used fluoride-containing dentifrices, and group 3 contained 14 patients who used dentifrices with natural ingredients.

### 3.2. Clinical Measurements

**Figure 2:**
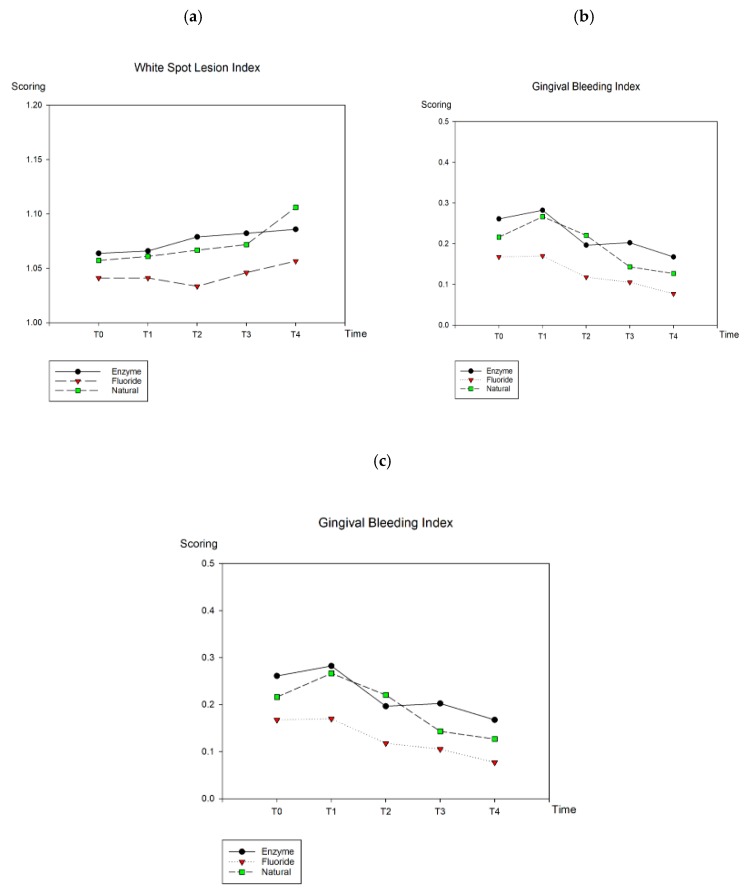
Comparisons of average scoring in three variables among the three dentifrices from T0 to T4: (**a**) WSL; (**b**) GBI; (**c**) VPI.

Table 1 and Figure 2 show the WSL, GBI, and VPI of all groups. No significant difference was noted in the WSL, GBI, and VPI among groups 1, 2, and 3 at baseline. At the end of the trial, no significant difference was noted in average WSL, GBI, and VPI among the three groups.

Within the groups, no significant difference in WSL was observed in groups 1 and 2 from baseline to 12 weeks of treatment, whereas a significant increase in WSL was noted in group 3 after the experiment.

The GBI scores indicated that all the groups showed improvement across the experiment. A significant decrease in GBI scores was observed from T1 to T4 in all groups. In addition, the GBI scores significantly decreased at T3 compared with T1 in group 3 and at T4 compared with T0 in group 2.

In group 1, VPI was significantly different between T0 and T4, T0 and T3, and T1 and T4, whereas it was significantly different between T0 and T4, T0 and T3, T1 and T4, and T1 and T3 in group 2. VPI in both groups 1 and 2 indicated significant decrease in plaque accumulation. No significant difference in VPI score was noted in group 3 from baseline to the 12th week, thus indicating no significant decrease in plaque accumulation.

### 3.3. Questionnaires

According to the questionnaire responses, the average tooth brushing frequency was 2–3 times/day (mean ± SD: 2.3 ± 0.7 times/day at baseline and 2.8 ± 0.8 times/day at the end of the trial), and no difference in tooth brushing frequency was observed among all groups at baseline and at the end of the experiment. Table 2 demonstrates the absence of difference among groups 1, 2, and 3 in the occurrence of oral ulceration, gum bleeding, tooth sensitivity, dry mouth, and bad breath before and after placement of the fixed appliance while using different dentifrices. The satisfaction levels regarding improvement in oral ulceration, gum bleeding, tooth sensitivity, dry mouth, and bad breath were nonsignificant among groups 1, 2, and 3 (Table 2).

## 4. Discussion

Dentifrices are daily oral care essentials, and the use of various types of dentifrices has previously been compared among orthodontic patients during treatment [16,17,18]. Our thorough search of relevant literature revealed that this is the first study to assess the effects of the use of enzyme-containing dentifrices without fluoride and different conventional dentifrices on orthodontic patients.

White spot lesions, the precursors to caries formation, are a common adverse effect of orthodontic treatment attributable to fixed orthodontic appliances [21] and prolonged exposure to bacterial plaque [22]. In the orthodontic population, white spot lesion occurrence ranges from 8.5% to 44% on anterior teeth and from 7.7% to 71% on posterior teeth [23]. The progression to clinically detectable white spot lesions can occur as early as 1 month after the placement of orthodontic appliances [24,25]; however, the formation of regular caries typically requires at least 6 months [26]. In this study, 1103 teeth were observed in 42 patients, and the rate of increase in white spot lesion occurrence was 2.3% in group 1, 1.6% in group 2, and 4.6% in group 3 after 3 months of fixed appliance placement, which was lower than the occurrence rate reported previously [23], indicating that the adverse effect of enamel demineralization was under control in the current study. Similar to the results of Hadler-Olsen et al. [27], the current study found no patient with a WSL score >2 at the end.

The benefits of topical fluoride in reducing demineralization and supporting remineralization are well recognized. Considerable research has been devoted to fluoride delivery methods that reduce or minimize enamel demineralization in orthodontic patients [28,29]. In this study, no significant increase in WSL was observed in group 2, similar to the previous finding, and white spot lesion prevention was also noted in group 1. The preventive effect of enzyme-containing dentifrices may be attributed to the reduction in the salivary levels of mutans streptococci and *Lactobacillus acidophilus*, according to Gudipaneni et al. [30]. With no addition of fluoride or enzyme into the dentifrice, the effect of the prevention of enamel decalcification was relatively weak in the patients in group 3, resulting in a significant increase in the amount of white spot lesions.

With conventional fixed orthodontic appliances, increased plaque accumulation occurs mainly on the labial or buccal surfaces of the teeth between the brackets or posterior bands and the gingival margins [31], and gingival inflammation is visible [32]. In the present study, the average GBI increased 3 weeks after bonding the brackets in all groups; however, the average GBI decreased significantly in the three groups at the end of the experiment. This result coincided with that of Moran et al. [15], which suggested that amyloglucosidase and glucose oxidase added to the toothpastes provides little or no more additional benefit to gingival health than does a conventional fluoride toothpaste. Furthermore, Etemadzadeh et al. [14] indicated that the inhibitory effect of the enzyme-containing toothpaste on plaque growth did not differ significantly from that of the conventional fluoride toothpaste, which is also consistent with the result of the current study. A possible explanation for these results is that the duration of the antimicrobial activity of the enzymes in vivo is short, even though the enzymes have some effects on plaque inhibition [33]. Although the result of the plaque-inhibiting effects in the current study conflicts those of previous studies [6,7,34], Adams et al. [12] suggested that toothpastes containing enzymes and proteins could enhance the ability of the natural salivary defense to promote an overall community shift, resulting in an increase in bacteria associated with gum health and a decrease in those associated with periodontal disease. According to the previous and present results, the effects of enzymes in the dentifrice on plaque change may be more related to the characteristics than to the amount. Nevertheless, studies [7,12,14,15,34] have added fluoride to the enzyme-containing dentifrices and compared them with conventional fluoride-containing dentifrices frequently used in non-orthodontic patients.

Soft tissue ulceration is another common side effect of orthodontic treatment [35]. Tenovuo [36] implied that whether the enzyme-containing dentifrices relieved the symptoms, including ulcers and dry mouth, because of antimicrobial proteins remains unknown. However, Donatsky et al. [37] suggested that the reduction effect of dentifrices containing amyloglucosidase and glucose oxidase on recurrent aphthous stomatitis was weak compared with that of a similar toothpaste without the enzymes. In the current questionnaire survey, no significant difference was noted in subjective assessment of oral condition during the experiment among all the groups. Ulceration on mucosa, which often occurs during orthodontic treatment or between treatment sessions around the brackets or end of wire [35], presumably contributed to this result. After 3 months of the experiment, no significant difference in user satisfaction regarding dentifrice use was reported among the three groups, and no patient chose the “more severe” option for the satisfaction level in the improvement category of the questionnaire. No adverse effect was reported during the experiment, but a few complaints regarding less foam in the dentifrice and texture that differed from their earlier texture preferences were received from several patients. Patients with elevated WSL scores during the experiment received intense follow-up and were asked to maintain good oral hygiene afterwards.

This clinical study had some limitations. First, the effect of a compound in a dentifrice is complicated by mechanical brushing, particularly in patients with fixed appliances. To minimize the effects of different mechanical cleaning processes on the results, uniformed brushing methods and frequency were established for the participants. Although the compliance of the volunteers was considered fair because 64% of the patients brushed their teeth three times a day and no significant difference in brushing frequency was seen among the patients in each group, the compliance of the patients could not be completely controlled, standardized, or measured. Moreover, patients involved in a clinical trial tend to maintain a higher level of oral hygiene (i.e., the Hawthorne effect), and thus, the result of the gingival condition may have been affected. Second, the sample size in this study was relatively small, which may have resulted in low statistical power. The smaller sample size made it more difficult to distinguish between a real effect and random variation. In addition, the trial duration was limited to the first 3 months of the orthodontic treatment, and hence the complete progression to demineralization and remineralization could not be easily detected in this short period. A future clinical trial with a larger sample size and an extended trial duration across the complete orthodontic treatment is warranted. Third, white spot lesions can be studied most effectively by performing a histosection of the tooth enamel, but this process involves sacrificing teeth and precludes a clinical study with live patients [38]. Because orthodontic treatment can affect the equilibrium of oral microflora by increasing the bacterial retention, a shift in bacterial composition and type can be expected [39]. To verify the ecology of oral microbiome associated with gingival health by using different dentifrices, the evaluation of species composition and level of supragingival plaque may be considered.

## 5. Conclusions

According to the results of this study, there were no significant differences between enzyme-containing dentifrices and conventional dentifrices in terms of white spot lesion prevention and plaque-reducing effects among orthodontic patients in the first 3 months of the treatment. Among the orthodontic patients using enzyme-containing dentifrices and fluoride-containing dentifrices, no significant increase in white spot lesions was observed in the first 3 months of the treatment, although a significant decrease in gingival bleeding and visible plaque were noted in the first 3 months of the treatment. No difference in satisfaction levels was noted between patients using enzyme-containing dentifrices and those using conventional dentifrices. Further research with a larger sample size and an extended duration across the complete orthodontic treatment is warranted.

## Figures and Tables

**Table 1:** Mean and standard deviation comparisons of white spot lesion index (WSL), gingival bleeding index (GBI), and visible plaque index (VPI) in groups 1, 2, and 3 through repeated measures ANOVA.

Dentifrice	T0	T1	T2	T3	T4	*p* Value
Enzyme						
WSL	1.06 ± 0.09	1.07 ± 0.09	1.08 ± 0.09	1.08 ± 0.09	1.09 ± 0.08	NS
GBI	0.26 ± 0.22	0.28 ± 0.27	0.20 ± 0.22	0.20 ± 0.14	0.17 ± 0.13	T1–T4 *
VPI	0.42 ± 0.21	0.37 ± 0.24	0.30 ± 0.22	0.25 ± 0.21	0.20 ± 0.20	T0–T4 *; T0–T3 *; T1–T4 *
Fluoride						
WSL	1.04 ± 0.06	1.04 ± 0.06	1.03 ± 0.06	1.05 ± 0.07	1.06 ± 0.08	NS
GBI	0.17 ± 0.13	0.17 ± 0.17	0.12 ± 0.14	0.11 ± 0.11	0.08 ± 0.11	T1–T4 *; T0–T4 *
VPI	0.34 ± 0.22	0.32 ± 0.26	0.24 ± 0.21	0.19 ± 0.18	0.17 ± 0.21	T0–T4 *; T0–T3 *; T1–T4 *; T1–T3 *
Natural						
WSL	1.06 ± 0.07	1.06 ± 0.07	1.07 ± 0.08	1.07 ± 0.06	1.11 ± 0.09	T0–T4 *
GBI	0.22 ± 0.23	0.27 ± 0.20	0.22 ± 0.19	0.14 ± 0.13	0.13 ± 0.11	T1–T4 *; T1–T3 *
VPI	0.28 ± 0.26	0.31 ± 0.24	0.26 ± 0.21	0.20 ± 0.17	0.18 ± 0.16	NS

NS = not significant, * *p* < 0.05. T0: baseline, prior to orthodontic appliance placement; T1: 3 weeks of treatment; T2: 6 weeks of treatment; T3: 9 weeks of treatment; T4: 12 weeks of treatment.

**Table 2:** The *p*-value for oral condition before and after the clinical trial and satisfaction regarding improvement among the three dentifrices.

Oral Condition	Enzyme	Fluoride	Natural	Satisfaction in Improvement
Oral ulceration	0.480	0.371	NA ^1^	0.424
Gum bleeding	0.617	0.480	NA	0.287
Tooth sensitivity	NA	NA	NA	0.123
Dry mouth	1	NA	1	0.282
Bad breath	0.134	1	NA	0.097

^1^ NA = not available.
